# A performance review of novel adiposity indices for assessing insulin resistance in a pediatric Latino population

**DOI:** 10.3389/fped.2022.1020901

**Published:** 2022-10-06

**Authors:** Mac B. McGraw, Lindsay N. Kohler, Gabriel Q. Shaibi, Lawrence J. Mandarino, Dawn K. Coletta

**Affiliations:** ^1^ Department of Physiology, The University of Arizona College of Medicine, Tucson, AZ, United States; ^2^ Center for Disparities in Diabetes, Obesity and Metabolism, The University of Arizona, Tucson, AZ, United States; ^3^ Exos, Phoenix, AZ, United States; ^4^ Center for Health Promotion and Disease Prevention, Arizona State University, Phoenix, AZ, United States; ^5^ Department of Medicine, Division of Endocrinology, The University of Arizona College of Medicine, Tucson, AZ, United States

**Keywords:** insulin resistance, adiposity indices, pediatrics, latino (Hispanic), obesity, type 2 diabetes

## Abstract

**Introduction:**

Body mass index (BMI) percentile or BMI adjusted for age and sex is the most common anthropometric index to monitor and assess obesity in children. However, the ability of BMI to accurately predict insulin resistance (IR) in youth is debated. Determining the best method to noninvasively measure IR in the pediatric population is especially important due to the growing prevalence of type 2 diabetes mellitus (T2DM), which is more likely to develop in people with IR. Therefore, this study analyzed the performance of BMI against newer anthropometric indices in assessing IR in a pediatric Latino identifying sample.

**Methods:**

We studied 127 pediatric Latino participants from the Arizona Insulin Resistance (AIR) registry and performed linear regression analyses between various measures of IR and adiposity indices, including body mass index (BMI), triponderal mass index (TMI), body adiposity index (BAI), pediatric body adiposity index (pBAI), a body shape index (ABSI), abdominal volume index (AVI), waist to height ratio (WtHR) and waist to hip ratio (WHR). Log transformations of each index adjusted for age and sex and IR were used for the linear regressions. Additionally, we generated receiver operating characteristics (ROC) from logistic regressions between HOMA-IR and HOMA2IR against the same indices.

**Results:**

Using the homeostatic assessment of insulin resistance (HOMA-IR), HOMA2IR, the quantitative insulin-sensitivity check index (QUICKI), fasting serum insulin, and FPG/FSI to measure IR, we showed that BMI adjusted for age and sex performs similarly to many of the newer indices in our sample. The correlation coefficients for pBAI [R2: 0.27, 95% confidence interval: 0.88–1.81, *p* < 0.001] and BMI [R2: 0.27, 95% confidence interval: 0.92–1.92, *p* < 0.001] were the highest for HOMA-IR. Similarly, pBAI [R2: 0.29, 95% confidence interval: 0.88–1.72, *p* < 0.001] and BMI [R2: 0.29, 95% confidence interval: 0.93–1.83, *p* < 0.001] were the highest for HOMA2IR. A similar trend was observed with QUICKI, FSI, and FPG/FSI. ABSI had the lowest *R*^2^ value for all measures of IR. Area under the curve (AUC) values for the receiver operating characteristics (ROC) for HOMA-IR and HOMA2IR support these conclusions.

**Conclusions:**

BMI adjusted for age and sex, despite its usage and simplicity, still stacks up well against newer indices in our Latino sample. Testing these indices across larger samples is necessary to generalize these findings and translate performance to adults.

## Introduction

Obesity is at epidemic proportions and is a growing concern in the United States and worldwide. The prevalence of childhood obesity in the United States is 19.7% and much higher at 26.2% in Hispanic/Latino children (1, 2). Childhood obesity is a serious health problem, putting children at risk for other diseases such as type 2 diabetes mellitus (T2DM). The increasing frequency of obesity and T2DM in children is a public health issue, and identifying those at risk is critical. Insulin resistance (IR), an underlying condition of obesity and T2DM, is often found in individuals with higher adiposity and is proportional to these increases in lipid mass (3). IR is especially threatening in children since the IR to T2DM transition is faster compared to adults (4).

IR is a reduced biological response to insulin and is particularly relevant at peripheral tissues such as skeletal muscle, white adipose tissue, and liver (5). When IR develops, the pancreas produces more insulin, resulting in hyperinsulinemia, to keep blood glucose levels within the standard range (5). Prediabetes occurs when the pancreas can no longer maintain a state of hyperinsulinemia to keep blood glucose levels within normal levels. As a result, β-cell dysfunction occurs, and IR worsens, resulting in T2DM. The IR observed in children and adolescents is important to identify and treat since pediatric patients with T2DM have faster β-cell function decline and poorer insulin sensitivity improvement with metformin than adults with T2DM (6).

The gold standard for quantifying IR is the hyperinsulinemic euglycemic clamp (7, 8); however, there are surrogate markers for assessing IR that are much simpler. These include the homeostasis model assessment of IR (HOMA-IR) derived from fasting plasma glucose (FPG) and fasting serum insulin (FSI), which has been validated against the clamp and is ideal when working with larger numbers of participants, particularly children and adolescents (9). However, there is no universal accepted parameter for defining IR in children and adolescents, and other indicators have been used including the quantitative insulin-sensitivity check index (QUICKI), fasting serum insulin (FSI), and FPG/FSI (10, 11).

Preventative screenings that detect precursor conditions for T2DM, such as IR, are critical for our pediatric populations, particularly Latino identifying youth, who are more likely to be obese and more at risk for other health-related diseases such as T2DM (12). Furthermore, screenings can guide referrals for intervention once impaired glucose tolerance is present (13). It is well established that an increased risk for IR is associated with obesity in pediatric patients. The National Institutes of Health and the World Health Organization use body mass index (BMI) to define obesity, and this index is well validated for assessing risk across many studies (14). However, BMI does not differentiate between fat and lean mass, nor does it portray the distribution of fat. In a meta-analysis of 37 studies analyzing 53,251 participants aged up to 18 years old, BMI correctly labeled people with excess adiposity only 73% of the time when compared to reference standard measurements *via* dual-energy x-ray absorptiometry (DXA) and similar methods (15). Also, it is especially limited for certain age ranges since BMI-z scores demonstrated poor associations with cardiometabolic risks in obese children (16). Moreover, the relationship between BMI and metabolic states varies among different pubertal stages, racial/ethnic groups, and health statuses being studied (17, 18). Several studies have noted superior associative scores of new adiposity indices, including triponderal mass index (TMI), pediatric body adiposity index (pBAI), a body shape index (ABSI), waist-to-height ratio (WtHR) and abdominal volume index (AVI) in measuring various metabolic parameters over age and gender-adjusted BMI and BMI-z scores in pediatric populations (16, 19–26). However, the novel adiposity indices mentioned above did not study children of Latino descent, which warrants an investigation.

This study aimed to analyze the association of novel body adiposity indices with various IR measures in a pediatric Latino identifying sample. In particular, our comparisons determined the efficacy of using BMI over newer indices when screening children and adolescents for IR using measures of HOMA-IR, HOMA2IR, QUICKI, FSI, and FPG/FSI. We hypothesize that these novel adiposity indices will better assess IR compared to BMI in our pediatric Latino identifying population.

## Materials and methods

### Participants

**Figure 1:**
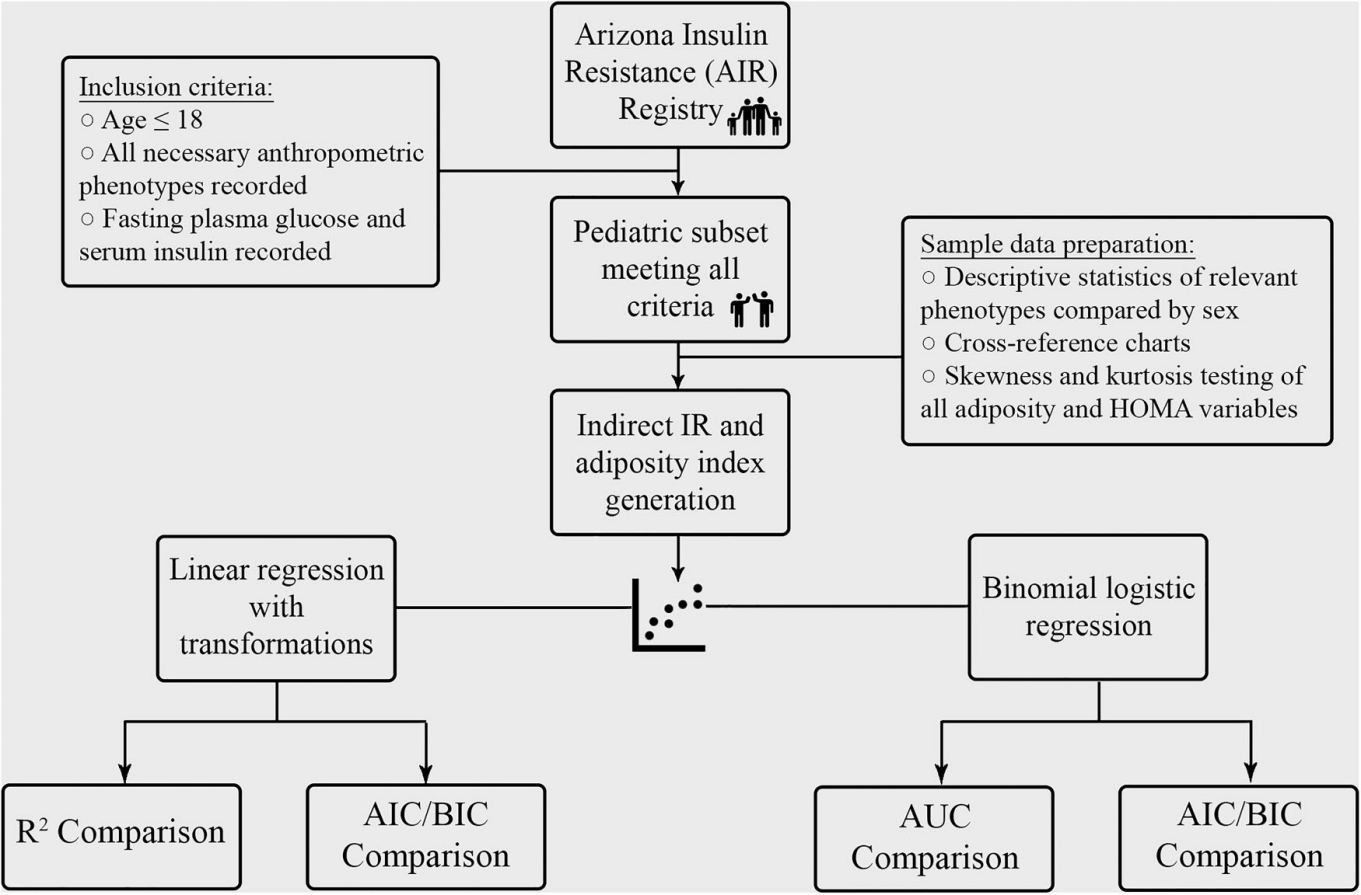
Outline of analysis.

Participants were selected from the Arizona Insulin Resistance (AIR) registry, as previously described by Shaibi *et al*. (27) Briefly, this registry included children and adults from the Latino identifying population in Arizona to establish a biobank for future investigation into metabolic health conditions in this community. Demographic, anthropometric, medical history and fasting metabolic panels were obtained from all participants. In total, 667 participants, of which 136 were ≤18 years old, were studied. Of the 136 children, 127 had complete metabolic data panels and were included in the analysis for the present study, as outlined in Figure 1.

The studies were reviewed and approved by The Institutional Review Board (IRB) at Arizona State University (ASU). ASU approved the initial AIR registry study under protocol #0804002873. At the time, written consent was obtained to bank serum, DNA, and RNA and to use de-identified data and biospecimens for future studies, like the one described herein. The University of Arizona approved the present study under protocol #1703255156. The present study was considered exempt by the ethics committee at the University of Arizona since it utilized de-identified information of previously consented banked data/samples. Therefore, we made no recontact with these participants. Written informed consent to participate in this study was provided by the participants' legal guardian/next of kin.

### AIR registry phenotypes used for analysis

The present study used participants' age, sex, BMI (kg/m^2^), height (cm), weight (kg), hip circumference (HC, cm), waist circumference (WC, cm), fasting plasma glucose (FPG, mg/dl) and fasting serum insulin (FSI, µU/ml). For the present study, we quantified IR using one of five surrogate indices: HOMA-IR: (FSI (µU/ml) × FPG (mg/dl)/405) (9), HOMA2IR using the HOMA2 online calculator (https://www.dtu.ox.ac.uk/homacalculator/), QUICKI (1/[log[FSI (µU/ml)] + log[FPG (mg/dl)]]) (28), FSI (µU/ml), and FPG (mg/dl)/FSI (µU/ml). We included HOMA2IR, a more recent method to measure IR that considers variations in hepatic and peripheral glucose resistance.

### Adiposity indices calculations

We used the following adiposity indices: body mass index (BMI), triponderal mass index (TMI), body adiposity index (BAI), pediatric body adiposity index (pBAI), a body shape index (ABSI), abdominal volume index (AVI), waist to height ratio (WtHR) and waist to hip ratio (WHR) in the regression analyses. Table 1 shows the equations and associated references describing these indices.

**Table 1:** Adiposity indices equations.

Index	Equation	Reference
Body mass index (BMI)	weight (kg)/height (m^2^)	(2, 29)
Triponderal mass index (TMI)	weight (kg)/height (m^3^)	(19)
Body adiposity index (BAI)	hip circumference (cm)/height (m^1.5^) - 18	(20, 21)
Pediatric body adiposity index (pBAI)	hip circumference (cm)/height (m^0.8^)−38	(30, 31)
A body shape index (ABSI)	waist circumference (m)/[BMI^2/3^ × height (m^1/2^)]	(22, 23)
Abdominal volume index (AVI)	[2WC (cm)^2^ + 0.7 (WC(cm)-HC (cm))^2^]/1000	(24, 25)
Waist to height ratio (WtHR)	waist circumference (cm)/height (cm)	(26)
Waist to hip ratio (WHR)	Waist circumference (cm)/hip circumference (cm)	(32)

### Statistical analysis

Statistical analyses were performed using STATA 14 (StataCorp, College Station, USA). Data were expressed as mean ± SD for descriptive statistics or mean ± SEM for linear regressions. Statistical significance of the difference between the participant characteristic data and adiposity indices by sex was measured using a non-paired Student *t*-test; *p* < 0.05 was considered statistically significant. Log transformation was used to create normally distributed data for the linear regression analysis. Normality was measured using skewness and kurtosis testing, with a significant *p* value indicating a statistically significant difference from a normal distribution (Supplementary Table S1). Linear regression analysis was performed between the adiposity indices and the surrogate measures of IR. Age and sex were included as covariates in the analysis. Furthermore, logistic regression analysis between HOMA-IR and HOMA2IR and the adiposity indices was performed. For the logistic regression analysis, we chose a HOMA-IR and HOMA2IR cutoff value of 2.5 based on a systematic review of HOMA-IR in pediatric populations (33). Receiver operating characteristic (ROC) and area under the curve (AUC) analysis were performed. Akaike information criterion (AIC) and Bayesian information criterion (BIC) postestimation values were calculated to compare index performance, with a lower AIC and BIC value suggesting a model that limits overfitting and maximizes goodness of fit (34). For the regression analyses, a *p* < 0.005 was considered statistically significant based on a Bonferroni's correction for the 10 adiposity measures used.

## Results

### Participants

Of the 127 pediatric participants included in the study, 64 were males, and 63 were females. The BMI distribution across the males and females is shown in Supplementary Table S2. Briefly, of the males: 0 were classified as underweight, 23 as healthy weight, 17 as overweight, and 24 as obese. Of the 63 females, 2 were classified as underweight, 36 as healthy weight, 14 as overweight, and 11 as obese. Data on the age distribution of participants by sex is shown in Supplementary Table S3. As shown in Table 2, height, weight, WC, HC and FPG (mg/dl) were significantly higher in males *vs.* females. Also, there was a significant difference between males and females for the AVI measure, as shown in Table 3. All other adiposity indices were not significantly different.

**Table 2:** Arizona Insulin Resistance (AIR) registry phenotypes by sex.

Phenotype	Male (*n* = 64)	Female (*n* = 63)	*p*
Age	14.3 ± 2.2	13.9 ± 2.7	NS
Height (cm)	165.5 ± 13.7	156.0 ± 11.1	<0.0001
Weight (kg)	70.1 ± 22.5	57.1 ± 20.5	<0.001
Waist circumference (cm)	86.9 ± 17.3	79.5 ± 17.2	<0.05
Hip circumference (cm)	99.7 ± 13.8	94.1 ± 15.0	<0.05
Fasting plasma glucose (mg/dl)	93.0 ± 5.8	89.6 ± 5.8	<0.01
Fasting serum insulin (µU/ml)	12.0 ± 11.0	10.6 ± 7.5	NS
HOMA-IR	2.8 ± 2.6	2.4 ± 1.7	NS
HOMA2IR	1.6 ± 1.3	1.4 ± 0.9	NS
QUICKI	0.35 ± 0.044	0.35 ± 0.038	NS
FPG/FPI	14.1 ± 11.0	13.1 ± 9.4	NS

Numbers represent the mean ± SD, NS, not significant.

**Table 3:** Adiposity indices by sex.

Index	Male (*n* = 64)	Female (*n* = 63)	*p*
BMI	25.1 ± 6.0	23.0 ± 6.8	NS
TMI	15.2 ± 3.4	14.7 ± 4.0	NS
BAI	28.9 ± 5.2	30.3 ± 5.8	NS
pBAI	28.6 ± 7.3	27.8 ± 8.6	NS
ABSI	0.079 ± 0.005	0.079 ± 0.005	NS
WtHR	0.52 ± 0.09	0.51 ± 0.10	NS
WHR	0.87 ± 0.08	0.84 ± 0.08	NS
AVI	15.8 ± 6.2	13.4 ± 6.4	<0.05

Numbers represent the means ± SD, NS, not significant; BMI, body mass index; TMI, triponderal mass index; BAI, body adiposity index; pBAI, pediatric body adiposity; ABSI, a body shape index; WtHR, waist to height ratio; WHR, waist to hip ratio; AVI, abdominal volume index.

### Linear regression for HOMA-IR, HOMA2IR, QUICKI, FSI, and FPG/FSI across adiposity indices

As shown in Tables 4, 5, the adiposity indices were regressed against HOMA-IR and HOMA2IR, respectively. The adiposity indices were also regressed against the QUICKI, FSI, and FPG/FSI, as shown in Tables 6–8, respectively. For the HOMA-IR, HOMA2IR, QUICKI, FSI, and FPG/FSI regressions, age and sex were included as covariates. Pertinent to the HOMA2IR analysis, to fit within the insulin and glucose ranges for the HOMA2 calculator, measurements above or below each glucose or insulin threshold were set to the maximum or minimum acceptable value. Five of the participants fasting insulin values of 2.76, 1.98, 2.8, 2.24, and 0.1 µU/ml were set to the minimum value of 2.9 µU/ml. One participant's fasting insulin value of 64.98 µU/ml was set to the maximum acceptable value of 57.6 µU/ml. As shown in Tables 4, 5, BMI and pBAI had the highest *R*^2^ value for the HOMA-IR and HOMA2IR. Also, ABSI had the lowest *R*^2^ value for the HOMA-IR and HOMA2IR. This trend is also consistent with the three other indicators of IR since BMI and pBAI had the highest *R*^2^ values when regressed against QUICKI, FSI, and FPG/FSI. ABSI consistently had the lowest *R*^2^ values across the other three IR surrogates, QUICKI, FSI, and FPG/FSI, and were not statistically significant.

**Table 4:** Linear regression between HOMA-IR and adiposity indices.

Variable	*R* ^2^	Variable Coefficient	Standard Error	CI	*p*	AIC	BIC
BMI	0.27	1.42	0.25	0.92–1.92	<0.001	257.67	269.04
TMI	0.26	1.43	0.26	0.91–1.94	<0.001	259.20	270.58
BAI	0.24	1.74	0.34	1.06–2.41	<0.001	262.52	273.90
pBAI	0.27	1.34	0.23	0.88–1.81	<0.001	256.75	268.13
ABSI	0.09	1.26	1.07	–0.86–3.38	NS	285.41	296.79
WtHR	0.25	1.80	0.34	1.12–2.48	<0.001	261.20	272.58
WHR	0.15	2.30	0.70	0.91–3.69	<0.001	276.16	287.54
WC	0.25	1.71	0.32	1.07–2.35	<0.001	260.75	272.13
HC	0.25	2.58	0.48	1.62–3.53	<0.001	260.21	271.59
AVI	0.25	0.88	0.17	0.55–1.21	<0.001	260.46	271.84

All variables were log base 10 transformed, age and sex were included as covariates in the model, CI, confidence interval; NS, not significant; AIC, Akaike information criterion; BIC, Bayesian information criterion; BMI, body mass index; TMI, triponderal mass index; BAI, body adiposity index; pBAI, pediatric body adiposity; ABSI, a body shape index; WtHR, waist to height ratio; WHR, waist to hip ratio; WC, waist circumference; HC, hip circumference; AVI, abdominal volume index.

**Table 5:** Linear regression between HOMA2IR and adiposity indices.

Variable	*R* ^2^	Variable Coefficient	Standard Error	CI	*p*	AIC	BIC
BMI	0.29	1.38	0.23	0.93–1.83	<0.001	232.17	243.54
TMI	0.28	1.38	0.24	0.91–1.85	<0.001	234.10	245.48
BAI	0.25	1.68	0.31	1.06–2.29	<0.001	238.14	249.52
pBAI	0.29	1.30	0.21	0.88–1.72	<0.001	231.60	242.98
ABSI	0.09	1.34	0.98	–0.61–3.28	NS	263.26	274.64
WtHR	0.27	1.76	0.31	1.14–2.38	<0.001	235.79	247.17
WHR	0.16	2.29	0.64	1.03–3.56	<0.001	252.52	263.90
WC	0.27	1.68	0.29	1.10–2.25	<0.001	235.09	246.46
HC	0.27	2.50	0.44	1.64–3.36	<0.001	235.03	246.41
AVI	0.27	0.86	0.15	0.57–1.16	<0.001	234.80	246.18

All variables were log base 10 transformed, age and sex were included as covariates in the model, CI, confidence interval; NS, not significant; AIC, Akaike information criterion; BIC, Bayesian information criterion; BMI, body mass index; TMI, triponderal mass index; BAI, body adiposity index; pBAI, pediatric body adiposity; ABSI, body shape index; WtHR, waist to height ratio; WHR, waist to hip ratio; WC, waist circumference; HC, hip circumference; AVI, abdominal volume index.

**Table 6:** Linear regression between QUICKI and adiposity indices.

Variable	*R* ^2^	Variable Coefficient	Standard Error	CI	*p*	AIC	BIC
BMI	0.26	−0.21	0.038	–0.29–(–0.14)	<0.001	–220.49	–209.11
TMI	0.25	–0.21	0.040	–0.29–(–0.13)	<0.001	–218.86	–207.48
BAI	0.23	–0.26	0.052	–0.36–(–0.16)	<0.001	–215.65	–204.27
pBAI	0.27	–0.20	0.036	–0.27–(–0.13)	<0.001	–221.56	–210.18
ABSI	0.09	–0.18	0.16	–0.50–0.14	NS	–193.57	–182.20
WtHR	0.24	–0.27	0.052	–0.37–(–0.16)	<0.001	–216.76	–205.38
WHR	0.15	–0.34	0.11	–0.55–(–0.13)	0.002	–202.29	–190.92
WC	0.24	–0.25	0.049	–0.35–(–0.16)	<0.001	–217.39	–206.01
HC	0.25	–0.39	0.073	–0.53–(–0.24)	<0.001	–218.30	–206.93
AVI	0.25	–0.13	0.025	–0.18–(–0.081)	<0.001	–217.66	–206.28

All variables were log base 10 transformed, age and sex were included as covariates in the model, CI, confidence interval; NS, not significant; AIC, Akaike information criterion; BIC, Bayesian information criterion; BMI, body mass index; TMI, triponderal mass index; BAI, body adiposity index; pBAI, pediatric body adiposity; ABSI, body shape index; WtHR, waist to height ratio; WHR, waist to hip ratio; WC, waist circumference; HC, hip circumference; AVI, abdominal volume index.

**Table 7:** Linear regression between FSI and adiposity indices.

Variable	R^2^	Variable Coefficient	Standard Error	CI	*p*	AIC	BIC
BMI	0.28	1.42	0.24	0.94–1.91	<0.001	249.51	260.89
TMI	0.27	1.43	0.25	0.93–1.93	<0.001	251.01	262.38
BAI	0.25	1.75	0.33	1.09–2.40	<0.001	254.36	265.74
pBAI	0.28	1.34	0.23	0.90–1.79	<0.001	248.44	259.82
ABSI	0.087	1.29	1.04	–0.78–3.35	NS	278.75	290.13
WtHR	0.25	1.81	0.33	1.15–2.47	<0.001	253.06	264.43
WHR	0.15	2.31	0.68	0.96–3.65	0.001	269.01	280.39
WC	0.26	1.71	0.31	1.09–2.33	<0.001	252.75	264.13
HC	0.26	2.57	0.47	1.65–3.49	<0.001	252.26	263.63
AVI	0.26	0.88	0.16	0.56–1.20	<0.001	252.47	263.84

All variables were log base 10 transformed, age and sex were included as covariates in the model, CI, confidence interval, NS, not significant; AIC, Akaike information criterion; BIC, Bayesian information criterion; BMI, body mass index; TMI, triponderal mass index; BAI, body adiposity index; pBAI, pediatric body adiposity; ABSI, a body shape index; WtHR, waist to height ratio; WHR, waist to hip ratio; WC, waist circumference; HC, hip circumference; AVI, abdominal volume index.

**Table 8:** Linear regression between FPG/FSI and adiposity indices.

Variable	R^2^	Variable Coefficient	Standard Error	CI	*p*	AIC	BIC
BMI	0.28	−1.42	0.24	−1.89–(–0.95)	<0.001	243.51	254.89
TMI	0.27	–1.43	0.25	–1.92–(–0.94)	<0.001	244.93	256.30
BAI	0.25	–1.75	0.32	–2.39–(–1.11)	<0.001	248.25	259.63
pBAI	0.29	–1.34	0.22	–1.78–(–0.91)	<0.001	242.29	253.66
ABSI	0.087	–1.32	1.02	–3.34–0.71	NS	273.85	285.23
WtHR	0.26	–1.81	0.33	–2.46–(–1.17)	<0.001	246.99	258.37
WHR	0.16	–2.31	0.67	–3.63–(–0.99)	<0.001	263.75	275.13
WC	0.26	–1.71	0.31	–2.31–(–1.10)	<0.001	246.86	258.24
HC	0.26	–2.56	0.46	–3.47–(–1.66)	<0.001	246.42	257.80
AVI	0.26	–0.88	0.16	–1.19–(–0.57)	<0.001	246.58	257.96

All variables were log base 10 transformed, age and sex were included as covariates in the model, CI, confidence interval; NS, not significant; AIC, Akaike information criterion; BIC, Bayesian information criterion; BMI, body mass index; TMI, triponderal mass index; BAI, body adiposity index; pBAI, pediatric body adiposity; ABSI, a body shape index; WtHR, waist to height ratio; WHR, waist to hip ratio; WC, waist circumference; HC, hip circumference; AVI, abdominal volume index.

### Logistic regression for HOMA-IR and HOMA2IR across adiposity indices

**Figure 2:**
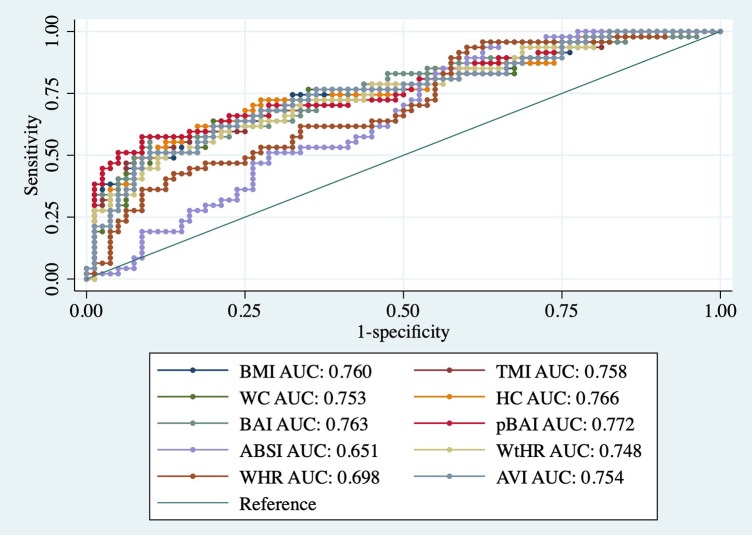
ROC curves of adiposity indices adjusted for age and sex against HOMA-IR. AUC, area under the curve; BMI, body mass index; TMI, triponderal mass index; BAI, body adiposity index; pBAI, pediatric body adiposity; ABSI, a body shape index; WtHR, waist to height ratio; WHR, waist to hip ratio; WC, waist circumference; HC, hip circumference; AVI, abdominal volume index.

**Figure 3:**
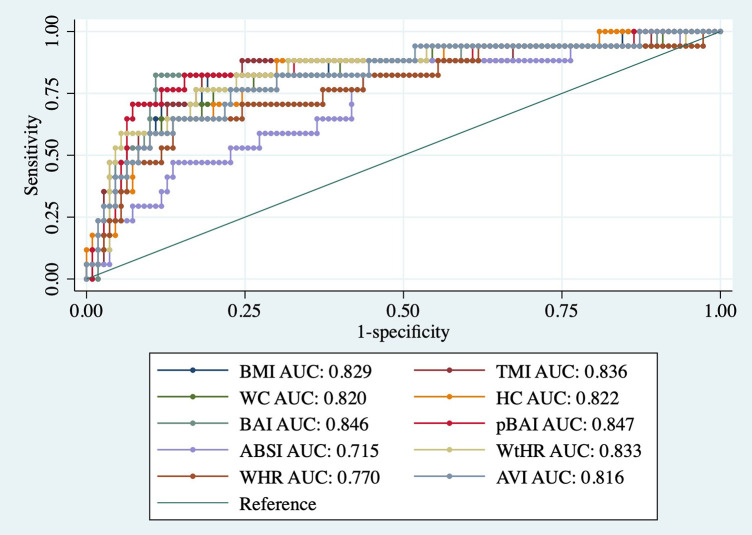
ROC curves of adiposity indices adjusted for age and sex against HOMA2IR. AUC, area under the curve; BMI, body mass index; TMI, triponderal mass index; BAI, body adiposity index; pBAI, pediatric body adiposity; ABSI, a body shape index; WtHR, waist to height ratio; WHR, waist to hip ratio; WC, waist circumference; HC, hip circumference; AVI, abdominal volume index.

As shown in Tables 9, 10, adiposity indices were regressed against HOMA-IR and HOMA2IR, respectively. HOMA2IR values were held to the same adjustments described for the linear regression methods to fit the minimum and maximum acceptable insulin and glucose values. As shown in Table 9, BMI and pBAI had similar odds ratios of 1.17 and 1.14, respectively with similar significance. As shown in Table 10, the similarities between these indices were consistent when regressed against HOMA2IR since the odds ratios for BMI and pBAI were 1.17 and 1.13, respectively with similar levels of significance. Receiver operating characteristic (ROC) curves were generated for both the HOMA-IR (Figure 2) and HOMA2IR (Figure 3) logistic regressions and compared by their area under the curve (AUC). As shown in Figure 2, BMI and the pBAI show a similar degree of sensitivity and specificity for IR since their AUC values were 0.760 and 0.772, respectively. As shown in Figure 3, there is a similarly strong performance between these indices regressed against HOMA2IR since BMI and pBAI have AUC values of 0.829 and 0.847, respectively.

**Table 9:** Logistic regression between HOMA-IR and adiposity indices.

Variable	Odds Ratio	Standard Error	CI	*p*	AIC	BIC
BMI	1.17	0.048	1.08–1.27	<0.001	144.94	156.31
TMI	1.28	0.083	1.12–1.45	<0.001	147.24	158.62
BAI	1.17	0.051	1.08–1.27	<0.001	148.65	160.03
pBAI	1.14	0.039	1.07–1.22	<0.001	143.83	155.21
ABSI	1.16	0.46	0.53–2.54	NS	165.48	176.86
WC	1.05	0.015	1.03–1.08	<0.001	148.70	160.08
HC	1.09	0.023	1.04–1.13	<0.001	143.85	155.22
WtHR	2.30	0.54	1.45–3.66	<0.001	150.44	161.82
WHR	1.67	0.42	1.02–2.73	NS	161.34	172.72
AVI	1.16	0.049	1.07–1.26	<0.001	148.66	160.04

Age and sex were included as covariates in the model. CI, confidence interval; NS, not significant; AIC, Akaike information criterion; BIC, Bayesian information criterion; BMI, body mass index; TMI, triponderal mass index; BAI, body adiposity index; pBAI, pediatric body adiposity; ABSI, a body shape index; WtHR, waist to height ratio; WHR, waist to hip ratio; WC, waist circumference; HC, hip circumference; AVI, abdominal volume index. ABSI values were multiplied by 100 and WtHR and WHR values were multiplied by 10 to scale values for logistic regression.

**Table 10:** Logistic regression between HOMA2IR and adiposity indices.

Variable	Odds Ratio	Standard Error	CI	*p*	AIC	BIC
BMI	1.17	0.050	1.07–1.27	<0.001	85.69	97.07
TMI	1.30	0.093	1.13–1.49	<0.001	85.42	96.80
BAI	1.20	0.059	1.09–1.32	<0.001	85.39	96.76
pBAI	1.13	0.039	1.06–1.21	<0.001	85.38	96.76
ABSI	1.88	1.09	0.60–5.89	NS	99.74	111.11
WC	1.06	0.018	1.02–1.09	<0.001	87.23	98.61
HC	1.07	0.022	1.03–1.12	<0.001	87.84	99.22
WtHR	2.70	0.75	1.56–4.66	<0.001	86.30	97.67
WHR	2.38	0.81	1.22–4.65	NS	94.21	105.58
AVI	1.15	0.048	1.06–1.25	0.001	88.18	99.56

Age and sex were included as covariates in the model. CI, confidence interval; NS, not significant; AIC, Akaike information criterion; BIC, Bayesian information criterion; BMI, body mass index; TMI, triponderal mass index; BAI, body adiposity index; pBAI, pediatric body adiposity; ABSI, a body shape index; WtHR, waist to height ratio; WHR, waist to hip ratio; WC, waist circumference; HC, hip circumference; AVI, abdominal volume index. ABSI values were multiplied by 100 and WtHR and WHR values were multiplied by 10 to scale values for logistic regression.

### Correlation of adiposity indices

**Figure 4:**
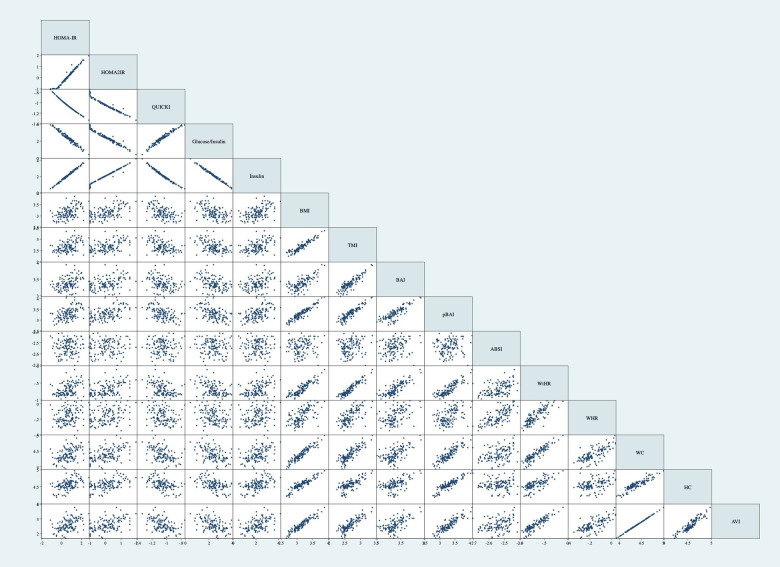
Graph matrix of adiposity indices. Correlations performed on log base 10 transformations. Correlations not adjusted for age and sex. BMI, body mass index; TMI, triponderal mass index; BAI, body adiposity index; pBAI, pediatric body adiposity; ABSI, a body shape index; WtHR, waist to height ratio; WHR, waist to hip ratio; WC, waist circumference; HC, hip circumference; AVI, abdominal volume index.

Table 11 shows the correlation matrix of the adiposity indices to determine the correlation coefficient between each measurement. Additionally, Figure 4 shows the graph matrix to visualize the correlation between these variables.

**Table 11:** Correlation matrix of adiposity indices.

	HOMA-IR	HOMA2IR	QUICKI	FPG/FSI	FSI	BMI	TMI	BAI	pBAI	ABSI	WtHR	WHR	WC	HC	AVI
HOMA-IR	1														
HOMA2IR	0.99	1													
QUICKI	−1.00	−0.98	1												
FPG/FSI	−0.99	−0.98	0.98	1											
Insulin	1.00	0.99	−0.99	−1.00	1										
BMI	0.50	0.52	−0.50	−0.51	0.51	1									
TMI	0.45	0.48	−0.45	−0.47	0.46	0.94	1								
BAI	0.36	0.38	−0.36	−0.39	0.38	0.72	0.88	1							
pBAI	0.50	0.53	−0.50	−0.52	0.51	0.94	0.92	0.83	1						
ABSI	0.04	0.06	−0.04	−0.05	0.05	0.09	0.16	0.21	0.12	1					
WtHR	0.43	0.46	−0.42	−0.45	0.44	0.89	0.94	0.83	0.87	0.48	1				
WHR	0.26	0.28	−0.25	−0.26	0.26	0.60	0.65	0.47	0.48	0.74	0.84	1			
WC	0.48	0.51	−0.48	−0.49	0.49	0.95	0.85	0.61	0.89	0.35	0.91	0.73	1		
HC	0.50	0.52	−0.50	−0.50	0.50	0.91	0.74	0.53	0.91	0.03	0.72	0.38	0.91	1	
AVI	0.49	0.51	−0.48	−0.49	0.49	0.95	0.85	0.61	0.89	0.33	0.90	0.72	1.00	0.92	1

Correlations performed on log base 10 transformations. Correlations not adjusted for age and sex. BMI, body mass index; TMI, triponderal mass index; BAI, body adiposity index; pBAI, pediatric body adiposity; ABSI, a body shape index; WtHR, waist to height ratio; WHR, waist to hip ratio; WC, waist circumference; HC, hip circumference; AVI, abdominal volume index.

## Discussion

The overarching goal of the present study was to compare various measures of adiposity to surrogates of IR in a sample of Latino identifying children and adolescents from the Arizona Insulin Resistance registry. We showed that, despite varied literature suggesting the inferiority of BMI as a reliable indicator of cardiometabolic conditions in pediatric populations, BMI adjusted for age and sex is still a comparatively strong indicator of IR. This is supported by a sample of 1,261 children between 6 and 10 years old since AUC values for abdominal skinfold thickness were not significantly different than BMI when regressed against HOMA-IR (35). Further, in a systematic review investigating TMI as a screening tool for IR in children and adolescents, the authors concluded that TMI did not outperform BMI (36). We conclude consistent findings with both papers, based on the higher linear regression correlation coefficients and low AIC and BIC values for HOMA-IR, HOMA2IR, QUICKI, FSI, and FPG/FSI. We confirmed these trends by demonstrating the consistently strong AUC values for logistic regression for these same indices against both HOMA-IR and HOMA2IR.

In this study, we used a linear regression analysis against multiple surrogate measurements of IR. We used linear regression for this analysis, since IR is a continuous variable representing a spectrum of varying responsiveness to insulin, rather than a binominal variable. Moreover, logistic regressions require a cutoff for insulin resistance, which would necessitate IR to be defined as a disease above a specific quantitative marker value. A review by Fox et al. demonstrates the problem with using a logistic approach, noting that across 298 analyzed papers, 51 different HOMA-IR cutoffs were used (37). Given this, our study only used logistic regressions as a confirmatory measure of our linear regression findings. IR was defined using HOMA-IR, HOMA2IR, QUICKI, FSI, and FPG/FSI since these indices only require FPG and FSI values. We chose multiple measures of IR for this present study, since there is no known universal accepted parameter for defining IR in children. Performing a euglycemic hyperinsulinemic clamp is the gold standard for directly assessing insulin sensitivity (7); however, these experiments are more time consuming and invasive since they require blood measurements over time in response to an insulin and glucose infusion (8). This procedure can be especially challenging in children, given the invasiveness and time commitment of the clamp. Using clamp data in lieu of our indirect IR indices may have led to different relative performance of the indices considered in this paper and should be kept in mind when interpreting the present findings.

Our study utilized various indices that merit discussion, including BAI and BAIp, which use height and hip circumference to estimate the body fat percentage of adults and adolescents. It was initially proposed by Bergman et al. as a replacement for BMI to better assess body adiposity (21). El Aarbaoui et al. used the same approach as Bergman to create the pediatric body adiposity index and found that it better assessed body fat percentage in a pediatric sample (30). However, in Brazilian children and adolescents, BAI tends to overestimate, and BAIp tends to underestimate actual body fat composition compared to the standard dual-energy DXA (31). Further, using an adult Mexican sample, the BAI less accurately indicated impaired fasting glucose than blood TG and the WHtR (20). In our sample, the BAIp was either the best or tied as the best index for indicating HOMA-IR or HOMA2IR. In tandem with the results of these other papers, our finding suggests that indices that assess one metric such as body fat percentage, can also evaluate other states, such as IR.

Additionally, we used ABSI, an index that uses waist circumference, BMI, and height as an alternative method to measure visceral fat area. It was created by Krakauer and Krakauer as an alternative index for assessing mortality risk (23). The models assessing ABSI and IR in our population did not reach statistical significance. This is consistent with the work of Liu et al., who demonstrate that ABSI was an inferior indicator of the visceral fat area than WC alone in a population of Chinese adults with T2DM (22). Various other indices were used, including TMI, which estimates body fat percentage more accurately than BMI in both non-Hispanic White and Italian children and adolescents, using DXA as a reference (16, 19). Also, TMI indicated more severe metabolic syndrome factors compared to BMI in an pediatric South Korean population, suggesting that BMI underestimates comorbidities deserving of attention and possible treatment (19). In our sample, TMI had a slightly lower linear correlation coefficient for TMI adjusted for the same parameters.

The WtHR uses WC and height, and WHR uses WC and HC as measures of adiposity. In a sample of 9,916 adult subjects in China that assessed seven total adiposity indices, WHtR was significantly associated with increased FPG, oral glucose tolerance test glucose values, hemoglobin A1c, and fasting insulin (26). The WtHR performed in the middle of the indices for linear regression analysis against HOMA-IR and HOMA2IR. In a sample of nondiabetic adult women from Peru, there was a positive correlation between the WHR and log of HOMA-IR values as well as WHR and serum insulin values (32). In our sample, the WtHR performed better than the WHR since WtHR had a higher correlation coefficient for both the HOMA-IR and HOMA2IR linear regression. In a sample of 240 children between 7 and 15 years old, WC alone was positively correlated with HOMA-IR (38). In a different multivariate regression of 471 adolescents aged between 10 and 18 years old, WC was also positively correlated with IR, as measured by HOMA-IR (39).

We also used AVI, which uses a combination of WC and HC to determine abdominal volume. Initially, the index was created to assess impaired glucose tolerance and T2DM (25). In an adolescent sample from Spain, this index and WC had the highest AUC value for determining metabolic syndrome compared to other anthropometric indices (24). The similarity in performance is consistent with our sample since both WC and AVI have the same correlation coefficient for both HOMA-IR and HOMA2IR linear regressions. Both indices also had similar AUC values for both HOMA-IR and HOMA2IR.

The change in relative performance between the indices in our sample compared to those using adult populations may be partially attributable to pubertal development, since height and weights vary during this time (40). For instance, WC alone better assesses T2DM risk in adults, but this is not as clear in pediatric populations (41). However, the performance of these indices varies across different population demographics. For example, in a meta-analysis of 10 studies evaluating the screening power of anthropometric indices in pediatric samples, WtHR was the index that performed better but not significantly different than WC or BMI (42).

Mid-upper arm circumference has been considered as a newer indicator for central obesity (43). This is especially promising for IR since it is an underlying feature of obesity, and visceral adipose tissue is correlated more strongly with IR than subcutaneous adipose tissue (44). Wrist circumference is another commonly used indicator of IR in children. In a sample of 477 overweight and obese children and adolescents, wrist circumference was better correlated with IR, as measured through HOMA-IR, than with BMI-SDS scores (45). Neither of these promising indices were measured or analyzed in this study.

A limitation of this study was the lack of pubertal stage data for our pediatric participants. Many papers try to combat the challenges with children by ranking the indices based on pediatric age ranges or pubertal stages. This may be due to the physiological changes in insulin sensitivity during development (4). Given that we did not have measures of puberty available, we opted to include all participants 8–18 years old and adjust for age in the analysis. However, even with this adjustment, this age range is large and is subject to changes in insulin resistance over time since insulin resistance has been shown to increase during puberty and fall back to pre-pubescent levels after puberty (46). As such, adjusting for age is not a valid substitute for Tanner staging since insulin resistance does not linearly rise with age. However, it is also important to consider that, in a sample of 777 children and adolescents, with children being defined as <10 years old, an association between IR and Tanner stage was only observed in children and not adolescents (39).

Additionally, a limitation is that these indices attempt to quantify phenotypes such as body fat percentage or visceral adiposity accumulation that are much better assessed using direct measures such as DXA scanners and bioelectrical impedance analysis (47). Thus, every index used in this paper should be used and interpreted with caution, knowing there are more definitive ways to measure the phenotypes being tested, including body fat percentage and IR.

Another major limitation of this study is the small sample size. Given the large age range of 8–18 years and the small composition of participants less than 12 years of age as seen in Supplementary Table S3, it is difficult to generalize the findings of these regressions beyond the sample. This is especially important when considering how insulin resistance changes with age since breaking up the analyses by age ranges and/or Tanner stages would provide more insight as to which adiposity indices are the best at different developmental stages. This was not possible given the unequal distribution of an already-small pool of participants at different age ranges. In addition, children and adolescents are grouped together in our sample rather than being analyzed independently due to the small proportion of participants under 10 years old, as seen in Supplementary Table S3. Since these age ranges are normally considered separately, future studies should validate our findings against larger samples of children and adolescents independently.

Despite the common concerns seen with BMI in pediatric populations, it is easy to measure and interpret in clinical settings due to its long history and ubiquitous use. Unlike other indices in this paper, BMI does not require any measurement beyond height and weight, making it easy to measure with little staff training. However, even with more invasive measurements, BMI still indicates IR well in some pediatric samples. In a sample of 543 children between 4 and 17 years old, BMI and WC better correlated with HOMA-IR than the Visceral Adiposity Index, a more invasive measure requiring blood triglyceride and high-density lipoprotein measurements (48). In a different sample of 777 children and adolescents, only BMI-SDS significantly correlated with IR in children and adolescents, with children being defined as <10 years old, compared to WC that only significantly correlates with IR in adolescents (39). Although the index pBAI did show slightly higher linear regression values with measures of IR, the challenge of training clinics to accurately measure hip circumference for a slight improvement in associative strength should be considered. Despite its age and simplicity, BMI adjusted for age and sex still performs well compared to newer indices in our Latino identifying children and adolescents from the AIR registry.

## Data Availability

The raw data supporting the conclusions of this article will be made available by the authors, without undue reservation.
