# Supplementary material for: A performance review of novel adiposity indices for assessing insulin resistance in a pediatric Latino population
Source: Front Pediatr. 2022 Oct 6;10:1020901. doi: 10.3389/fped.2022.1020901 (PMC9582658; doi:10.3389/fped.2022.1020901)
Supplement: Supplementary file 1 [file DataSheet1.docx]

Supplementary Material

**Supplementary Table 1.** Skewness and kurtosis testing for normality across variables

| **Variable** | **Untransformed Chi** | **Untransformed p** | **Transformed* Chi** | **Transformed p** |
| --- | --- | --- | --- | --- |
| BMI | 20.83 | < 0.0001 | 4.76 | NS |
| TMI | 25.84 | < 0.0001 | 8.41 | < 0.05 |
| BAI | 23.56 | < 0.0001 | 6.52 | < 0.05 |
| pBAI | 25.36 | < 0.0001 | 4.59 | NS |
| ABSI | 4.25 | NS | 4.27 | NS |
| WtHR | 21.66 | < 0.0001 | 8.65 | < 0.05 |
| WHR | 7.24 | < 0.05 | 7.97 | < 0.05 |
| WC | 15.27 | < 0.001 | 2.52 | NS |
| HC | 8.63 | < 0.05 | 1.83 | NS |
| AVI | 36.91 | < 0.0001 | 2.61 | NS |
| HOMA-IR | 65.38 | < 0.0001 | 0.19 | NS |
| HOMA2IR | 46.47 | < 0.0001 | 3.99 | NS |
| QUICKI | 6.18 | 0.0454 | 1.40 | NS |
| FSI | 61.74 | < 0.0000 | 0.29 | NS |
| FPG/FSI | 32.07 | < 0.0000 | 0.45 | NS |

*Log base 10 transformed, NS = not significant. BMI = body mass index, TMI = triponderal mass index, BAI = body adiposity index, pBAI = pediatric body adiposity, ABSI = body shape index, WtHR = waist to height ratio, WHR = waist to hip ratio, WC = waist circumference, HC = hip circumference, AVI = abdominal volume index, QUICKI = quantitative insulin-sensitivity check index, FSI = fasting serum insulin, FPG/FSI = fasting plasma glucose/fasting serum insulin.

**Supplementary Table 2.** BMI distribution of Pediatric Arizona Insulin Resistance (AIR) Registry Participants

| **BMI category** | **Male (n=64)** | **Female (n=63)** | **Total (n=127)** |
| --- | --- | --- | --- |
| Underweight | 0 | 2 | 2 |
| Healthy weight | 23 | 36 | 59 |
| Overweight | 17 | 14 | 31 |
| Obese | 24 | 11 | 35 |

**Supplementary Table 3.** Age distribution of Pediatric Arizona Insulin Resistance (AIR) Registry Participants

| **Age range** | **Male (n=64)** | **Female (n=63)** | **Total (n=127)** |
| --- | --- | --- | --- |
| 8-10 | 3 | 8 | 11 |
| 10-12 | 6 | 4 | 10 |
| 12-14 | 17 | 24 | 41 |
| 14-16 | 22 | 9 | 31 |
| 16-18 | 16 | 18 | 34 |
